# Identifying With How We Are, Fitting With What We Do: Personality and Dangerousness at Work as Moderators of Identification and Person–Organization Fit Effects

**DOI:** 10.5964/ejop.v15i2.1518

**Published:** 2019-06-07

**Authors:** Emmanouela Mandalaki, Gazi Islam, Urszula Lagowska, Camila Tobace

**Affiliations:** a Department of Organizations, University of Los Andes, Bogota, Colombia; b Department of People, Organizations and Society, Grenoble Ecole de Management, Grenoble, France; c Brazilian School of Public and Business Administration, Fundação Getulio Vargas, Rio de Janeiro, Brazil; d Department of Administration, Insper Institute for Education and Research, São Paulo, Brazil; Webster University Geneva, Geneva, Switzerland

**Keywords:** person-organization fit, identification, dangerousness, job performance, neuroticism

## Abstract

Using a sample drawn from a Brazilian electric company exposing employees to both dangerous and non-dangerous working conditions, the current study provides evidence on the differential underlying mechanisms guiding the relationships of organizational identification and person-organization-fit (P-O fit) with job performance. We suggest that despite their relatedness in current literature, organizational identification operates as a largely self-centered process and P-O fit as a predominantly context-dependent one, leading to distinct work-related processes deriving from each construct. Our findings suggest that P-O fit serves as a pathway through which job identification induces job performance. In this mediating path, personality and in particular neuroticism, hinders the effects of identification, whereas job dangerousness, a contextual factor, undermines work-related effects of perceived environmental congruence (P-O fit). Discussing these results, we provide novel insights on the distinct mechanisms driving organizational identification, P-O fit and their contingencies.

Organizational identification, defined as perceived belongingness to the organization (Tajfel, 1978), is a key driver of employee motivation and performance (e.g., Ashforth, Harisson, & Corley, 2008; Brickson, 2012; Riketta, 2005; van Knippenberg, 2000). Some research suggests that identification effects depend on how one sees one’s relation to the collective, which involves the cognitive activation of group-related constructs by workers (Ashmore, Deaux, & McLaughlin-Volpe, 2004; Simon & Klandermans, 2001; van Knippenberg, 2000). Among these constructs is the subjective sense of perceived alignment with the organization, or person-organization fit (e.g., Edwards, 2008). Even though the latter overlaps conceptually with identification (Ashforth et al., 2008), it remains distinct in that it involves perceptions of an environmental match between the individual and the collective (Edwards, 2008; Edwards, Cable, Williams, Lambert, & Shipp, 2006). On the other hand, identification mainly centers on self-definition (Ashforth et al., 2008; Postmes & Jetten, 2006); driven by categorization processes and attachment to the collective (Ashmore et al., 2004; Simon & Klandermans, 2001).

Despite differentiations in the literature between identification and subjective organizational fit perceptions (e.g., Nolan & Harold, 2010; Pratt, 1998), a clear understanding of how they differ conceptually and empirically to affect workplace outcomes remains elusive. Given that identification and P-O fit share similar correlates, such as organizational values, goals and objectives (Ashforth et al., 2008; Besharov, 2014) and that they both involve relations between the self and the context, they can be easily conflated in practice. To understand how identification processes interface and differ with subjective P-O fit perceptions, it is crucial to disentangle the distinct ways in which they drive employee outcomes, such as job performance.

Responding to calls for more research on factors affecting P-O fit and identification-related processes (He & Brown, 2013; Kristof-Brown, Zimmerman, & Johnson, 2005), we empirically test the distinct underlying mechanisms guiding work-related outcomes of each of the two constructs (Cha, Chang, & Kim, 2014; Chen, Chi, & Friedman, 2013; Edwards, 2008; van Knippenberg, 2000). Identification centers on perceptions of oneself as integral to the organization and perceptions of the organization as integral to oneself (Albert, Ashforth, & Dutton, 2000; Ashforth et al., 2008; Simon & Klandermans, 2001), while subjective P-O fit reflects the consideration of the self vis-a-vis the organizational context (Kristof-Brown et al., 2005). Thus, we would expect job-related identification effects to depend more on identifying personal characteristics, like personality, whereas fit effects to be conditional upon situational factors affecting motivation and job performance, such as dangerousness at work (Sousa-Poza & Sousa-Poza, 2000).

First, we investigate how personality characteristics -namely agreeableness and neuroticism- guide organizational identification’s work-related effects (Schneider, 2007). Second, building on literature citing contextual attributes as drivers of P-O fit at work (e.g., Edwards, 2008; Jansen & Kristof-Brown, 2006), we discuss how dangerousness, an extreme contextual characteristic, drives perceptions of subjective P-O fit effects on work outcomes (Lambert, Hogan, Paoline, & Clarke, 2005; Price, 1997). Our results extend literature discussing the role of personality characteristics in shaping work-related outcomes of organizational identification (e.g., Nicol, Rounding, & MacIntyre, 2011; Schneider, 2007; Swann, 1990; Vignoles, Regalia, Manzi, Golledge, & Scabini, 2006), while examining contextual demands influencing P-O fit outcomes.

We propose a conceptual model linking personality characteristics with identification perceptions, and contextual characteristics with P-O fit perceptions, in affecting job performance. We argue that *who one is and how one sees themselves in relation to the collective* (i.e., identification) is particularly sensitive to personality factors, whereas *how one fits* (i.e., P-O fit), as a result of their perceived organizational identification and relation to the context, shapes job performance depending on contextual factors. Our results suggest that neuroticism negatively guides the relationship between identification and performance, while dangerousness at work undermines links between perceptions of subjective P-O fit and job performance. By shedding light on individual vs. contextual factors jointly driving the effects of identification and P-O fit on job performance, we contribute to current debates calling for a better understanding of the underlying processes driving work-related outcomes of the two constructs (Nolan & Harold, 2010; Pratt, 1998). We discuss the implications of our research for theory and for managerial practice.

## Identification and P-O Fit as Distinct Workplace Processes

### Identification With Organization, P-O Fit and Job Performance

Understanding the distinct psychological bases of identification vis-a-vis other states of employee organizational embeddedness has led scholars to explore the role of social identity in organizations (cf. Mael & Ashforth, 1995; Ashforth et al., 2008; Brickson, 2012; van Knippenberg & Hogg, 2003). Social identification relates to self-definition in terms of group membership, reflecting belongingness and emotional attachment to the group (Mael & Ashforth, 1992; Tajfel, 1978). According to Dutton, Dukerich, and Harquail (1994, p. 242), organizational identification is the “psychological attachment that occurs when members adopt the defining characteristics of the organization as defining characteristics of themselves.” Even though scholars discuss the collective aspect of identity (Ashmore et al., 2004; Simon & Klandermans, 2001), literature suggests that identity remains largely dependent on the self; since it is the individual that evaluates the group and their relationship with it (Eidelman & Silvia, 2010; Vignoles, Schwartz, & Luyckx, 2011).

A growing body of organizational behavior literature has identified links between identification at work and other organizational constructs like participation, collective effort, and job performance (e.g., Ashforth et al., 2008; Kramer, 2006; van Knippenberg, 2000). Particularly, van Knippenberg (2000) argued that identification with the work environment is positively related to both motivation as well as task and contextual performance. Similarly, literature cites identification both as a moderator and mediator guiding the relationship between several antecedents and job-related outcomes, like job performance (e.g., Blader, 2007; Chen et al., 2013). The latter suggests that identification serves as a proximal cause of job outcomes.

Tajfel (1982) posits that identification involves three basic constituents: a cognitive component, an evaluative component, and an emotional one. More recent accounts cite emotions as an important element guiding organizational identification development (Hermans & Dimaggio, 2007). Considering the group-affiliative and emotional nature of identification, focusing on group-oriented personality characteristics, like agreeableness, and emotionally laden ones, such as neuroticism, as underlying mechanisms potentially driving its work outcomes, makes sense in this context.

However, because identification refers to self-perception in a group context, its relation to specific aspects of the workplace is likely mediated by more proximal comparisons between the self and the context. Further, the self-oriented aspect of identification (Postmes & Jetten, 2006) with regards to the collective, might explain its points of differentiation from other overlapping constructs, like P-O fit, which refers more directly to the organizational environment (Edwards et al., 2006). In fact, a substantial body of literature has discussed the relationship between identification and P-O fit (as opposed to other similar notions), while the conceptual differences between the two have raised an interesting debate in academic literature (e.g., Chatman, 1989; Werbel & Gilliland, 1999). Exploring P-O fit vis-a-vis identification work-related outcomes enables us to identify distinct dynamics guiding the two to induce job performance.

### Person–Organization Fit

Werbel and Gilliland (1999, p. 217) define P-O fit as “the congruence of employees’ needs, goals, and values with organizational norms, values, and reward systems.” The latter considers fit in terms of a “match” between the individual and the organizational environment, depending on an evaluation of how one’s individual characteristics, needs and perceived identification match with those of the context. A growing body of organizational behavior literature cites the positive effect of P-O fit on employee commitment, satisfaction and feelings of competence and identifies positive P-O fit effects on organizational performance and effectiveness (Edwards, 2008; Jansen & Kristof-Brown, 2006; O’Reilly, Chatman, & Caldwell, 1991; Werbel & Gilliland, 1999).

Literature also cites some conceptual similarities between organizational identification and subjective P-O fit (Foreman & Whetten, 2002; Meyer, Becker, & Vandenberghe, 2004). However, although they both consider employees’ identity and one’s relation to the environment, they also demonstrate important conceptual differences (Ashforth et al., 2008). For instance, Pratt (1998) notes that fit is a broader concept relying on contextual assessments including both similarities and congruities based on difference or complementarity, whereas identification is a self-related judgment derived from group membership (Ashforth et al., 2008). Identification should thus relate to the context to the extent that the latter internally guides self-image formation. By contrast, in P-O fit assessments consider evaluation of oneself but remain largely context-dependent, whereby the person and the organization remain two different entities (Edwards et al., 2006). Such assessments can be thus re-produced in other organizational contexts, as long as the person perceives a relational match with the environment.

With regards to the relationship between these two concepts, literature suggests that P-O fit may be affected by organizational identification. Specifically, it has been shown that organizational identification satisfies meaning creation needs, leading to group cohesiveness and commitment to organizational values (Hogg, 2003; Hogg & Mullin, 1999); the latter also being an antecedent of P-O fit (Edwards, 2008). Further, Tajfel (1982) characterizes identification as a cognitive state whereby the person develops awareness of group membership to proceed to an evaluative state enhancing congruence with their working environment. Thus, while self-centered in nature, identification should be considered when individuals evaluate perceptions of subjective P-O fit. As such, job identification and subjective P-O fit are conceptually related, with identification providing a basis for fit.

In terms of outcomes, P-O fit enhances work motivation and commitment (Chatman, 1989; O’Reilly et al., 1991); psychological states also regarded as outcomes of identification leading to job performance. However, unlike identification, which has been cited to indirectly induce job performance (van Knippenberg, 2000), P-O fit is argued to have a direct link to it (Edwards, 2008; Werbel & Gilliland, 1999). The latter might imply that P-O fit could result from identification to influence job performance.

Considering the above and the fact that identification is a self-oriented psychological process shaping one’s self-image as part of a group, it should lead to performance to the extent that it triggers more proximal processes, such as subjective P-O fit. In other words, while not reflecting any urge for action in itself, identification should guide the development of feelings of congruence, in turn leading the person to perform better at work. That said, identification with the organization should lead to job performance through P-O fit. Thus:

**Hypothesis 1**: *P-O fit mediates the relationship between organizational identification and job performance, such that organizational identification leads to job performance through subjective P-O fit*.

Given the above, identification and subjective P-O fit are best seen as working together to produce work outcomes. However, due to their conceptual differences, we would expect distinct processes underlying their effects. As mentioned above, we examine personality and contextual factors to enhance our understanding of the differential underlying patterns guiding the work-related outcomes of identification and P-O fit, respectively.

### Personality and Contextual Characteristics

Given the conceptual differences identified above, we would expect the effects of identification, as a mainly self-oriented process, to be affected by identifying personal characteristics, like personality traits. Conversely, we would expect those of P-O fit, as a mainly context-laden process, to be more dependent on situational factors. Research has investigated the dynamics guiding each of these two components (e.g., Steele, 1988; Swann, 1990; Vignoles et al., 2006) and has established the importance of personality characteristics for adaptation, performance, interpersonal relations and identification with the working environment (e.g., Cheek & Briggs, 1982; Edwards, 2008).

A personality factor which has long been studied and suggested as a driver of job performance is conscientiousness (Resick, Baltes, & Shantz, 2007; Salgado, 1997). However, only a few studies discuss the effects of other personality characteristics, like agreeableness and neuroticism (John & Srivastava, 1999), on group identification and performance (Barrick & Mount, 1991; Johnson, Morgeson, & Hekman, 2012; Mount, Barrick, & Stewart, 1998). Responding to calls for more research of how personality characteristics drive identification processes at work (He & Brown, 2013), we focus on two understudied personality traits in relation to organizational identification—agreeableness and neuroticism—due to their conceptual similarities with the group-affiliative and emotional aspects of identification, respectively.

Considering that identification involves evaluations of the self and its relation to the collective as well as the fact that agreeableness, as a personality characteristic, involves pro-social attitudes towards interpersonal relationships and cooperativeness within groups (Hogan & Holland, 2003), agreeableness should be relevant to organizational identification’s job-related effects. Similarly, given that identification reflects an emotional investment in group membership (Tajfel, 1982), we would expect neuroticism, an emotionally laden personality characteristic (Denson, Spanovic, & Miller, 2009), to guide identification effects. Particularly in contexts of high personal or emotional tension (such as stressful work; Chen & Spector, 1992; Lambert, Kelley, & Hogan, 2012), we would expect neuroticism to influence identification effects because of its association with high emotional arousal (Denson et al., 2009).

Conversely, compared to personal characteristics, situational characteristics are likely to be more closely related to perceptions of congruence with the working environment (Jansen & Kristof-Brown, 2006). Thus, we would expect dangerousness, an understudied, extreme contextual factor, to shape P-O fit effects at work. Our examination of dangerousness allows the evaluation of context in a way that is salient and important in many sectors of work and blue-collar populations, which are likely to face major safety hazards at work; especially in less developed economies (ILO, 2003).

Indeed, satisfying job security needs has traditionally been cited as a basic condition leading to satisfaction at work (Herzberg, 1974). Moreover, recent research cites lack of job safety as a job stressor undermining satisfaction and job performance (Pasupuleti, Allen, Lambert, & Cluse-Tolar, 2009; Sousa-Poza & Sousa-Poza, 2000). Based on the above, exploring job dangerousness as a situational factor seems particularly relevant in the context of job outcomes.

### Agreeableness and Neuroticism

Agreeableness involves personal qualities of cooperativeness, social adaptability, and interpersonal sensitivity (John & Srivastava, 1999; Oh & Berry, 2009) with tendencies to act pro-socially (Hogan & Holland, 2003). Much like with organizational identification (Ashforth et al., 2008), literature cites a positive association between agreeableness and compliance (Organ & Ryan, 1995) or interpersonal facilitation (Hurtz & Donovan, 2000). The latter implies that agreeableness and identification might interact to enhance environmental congruency states. Similarly, recent research posits that emotional regulation and identification enhance agreeable individuals’ perceived organizational well-being (Larsen, 2000), suggesting that agreeableness might also explain links between organizational identification and P-O fit perceptions.

Yet, consistent with research citing very weak links between agreeableness and job performance (Barrick & Mount, 1991; Judge, Livingston, & Hurst, 2012), we would not expect agreeableness to affect job performance directly. Instead, considering the group-oriented and affiliative aspect of agreeableness (Witt, Burke, Barrick, & Mount, 2002), and its connection to identification, which also focuses on establishing belongingness to a group (Hermans & Dimaggio, 2007; Mael & Ashforth, 1992), agreeableness is likely to influence work outcomes of identification.

Some research suggests that agreeable individuals tend to experience adverse affective reactions in situations of conflict at work (Moskowitz & Coté, 1995), while they perform better in jobs requiring high cooperation with others (Witt et al., 2002). Agreeableness also moderates the relationship between organizational interaction beliefs and feelings of work congruence (Ehrhart, 2006), implying that it might also enhance states of P-O fit. As such, we would expect the cooperative and conflict avoidance tendencies associated with agreeableness to amplify the impact of organizational identification on perceived subjective fit, such that highly identified individuals at higher levels of agreeableness would reach states of higher fit as opposed to individuals at lower levels of agreeableness.

Based on the above, we hypothesize that:

**Hypothesis 2:** *Agreeableness moderates the relationship between organizational identification and P-O fit, such that the effect of organizational identification on P-O fit will be higher for agreeable as opposed to less agreeable individuals*.

While agreeableness reflects relational qualities between individuals, neuroticism, the opposite of emotional stability, involves negative affective experiences. Neuroticism is linked to tension, anxiety (Costa & McCrae, 1992; John & Srivastava, 1999), irritability, anger, depression, and emotional vulnerability (John & Srivastava, 1999) and is thought to indirectly affect work-related outcomes, like job performance (e.g., Barrick & Mount, 1991; Judge & Bono, 1999). Further, the emotional distress associated with neuroticism is negatively associated with self-concept clarity and social conformity attitudes (Froming & Carver, 1981; Scheier, Carver, & Gibbons, 1979). Finally, typical for neurotic individuals, low self-esteem negatively impacts compliance attitudes (Brockner, 1984).

Notably, literature suggests broad links between neuroticism and identification (Johnson et al., 2012). As discussed above, organizational identification is driven by the need to establish belongingness to a group and has positive implications for performance (Mael & Ashforth, 1992). However, given that neurotic people have low self-esteem (Eysenck, 1990; Watson & Clark, 1984), demonstrate broad tendencies of low social conformity (Furr & Funder, 1998), react more negatively to critical feedback (Larsen & Ketelaar, 1989) and have ill-defined self-concepts (Judge, Eres, Bono, & Thoresen, 2002), we would expect the positive work-related outcomes of high identification to be undermined at higher levels of neuroticism. This may be explained by the fact that as a result of their unstable emotional structure, neurotic individuals tend to take things more personally under conditions of high identification, with possible implications for job performance. As such, in situations of high organizational identification, the emotional instability associated with neuroticism would likely reduce work-related identification effects. Consequently, even though neuroticism does not directly affect job performance (e.g., Barick & Mount, 1991), it is likely to shape it indirectly. Thus:

**Hypothesis 3:** *Neuroticism moderates the relationship between organizational identification and job performance, such that the effects of organizational identification on job performance will be lower at high levels of neuroticism as opposed to low levels of neuroticism*.

### Dangerousness at Work

Literature has often discussed the effects of contextual and situational factors on P-O fit (Jansen & Kristof-Brown, 2006). Considering the context-laden nature of P-O fit (Jansen & Kristof-Brown, 2006; Resick et al., 2007), we explore dangerousness at work, a relatively understudied extreme situational characteristic, which is argued to impact job performance and P-O fit effects on work-related outcomes (Demerouti, 2006; Lambert et al., 2005; Viscousi, 1979). Dangerousness at work is defined as the extent to which a job can be physically dangerous and/or unsafe (Kim, 1996). Some scholars see dangerousness and work hazards as job stressors (Lambert et al., 2005; Pasupuleti et al., 2009; Price, 1997; Triplett, Mullings, & Scarborough, 1999), although this point is controversial (Viscousi, 1979) since it might depend on individuals’ profiles. For instance, for individuals high in sensation seeking, who usually display negative attitudes towards safety in the workplace (Henning et al., 2009), dangerousness might be faced as less of a negative aspect of work. However, we argue that in mundane physical work, such as construction or industrial work, dangerousness levels would likely be experienced as something to be avoided and would likely be counterproductive to work motivation and performance. Indeed, literature suggests that dangerousness might adversely affect the ability to complete work tasks (Price, 1997), and discusses its negative implications for job satisfaction and performance (Pasupuleti et al., 2009; Sousa-Poza & Sousa-Poza, 2000).

Similarly, research suggests that dangerousness at work enhances conflict, frustration and work stress, and impacts moral and affective job commitment (Lambert et al., 2005; Triplett et al., 1999). As such, reflecting risky and perilous working conditions, dangerousness has implications for work outcomes (Kim, Cyphert, & Price, 1995; Sousa-Poza & Sousa-Poza, 2000) resulting from subjective perceptions of congruence with the working environment (Lambert et al., 2012). Thus, we argue that dangerousness should negatively underlie P-O fit effects (potentiate misfit) (McGrath, 1976) on job performance. As such:

**Hypothesis 4:** *Dangerousness at work moderates the relationship between P-O fit and job performance, such that in non-dangerous jobs, the perceived P-O fit would lead people to exhibit better performance, as opposed to (highly) dangerous jobs*.

## Method

### Sample and Procedure

The sample of the study consists of electrical workers working under different levels of dangerous working conditions, in a medium sized firm in the interior of Sao Paulo state, in Brazil. One hundred and nine out of 508 employees participated in the study (21%). The survey was given to the HR department, which distributed it to the supervisors and asked them to apply it anonymously upon employees’ arrival at work. All scales were translated and back translated from English to Portuguese by native speakers. On average, survey participants had completed high school (*M* = 1.76, in a scale 1–3, where 1 = primary school, 2 = high school, 3 = university degree), 43 months of employment in the firm, and earned an average monthly salary of 1,642 Brazilian Reais (approximately 400 U.S. dollars).

To date, most research evidence on identification and P-O fit effects has been based on white-collar worker samples from the global North. We complement the latter approach, by studying a blue-collar sample drawn from an objectively dangerous working environment in the interior of Brazil. Doing so allows us to provide novel perspectives on the relationships that we study.

### Measures

#### Identification With Organization

 We measured organizational identification using the 6-item organizational identification scale by Mael and Ashforth (1992). The scale’s reliability in the current study is acceptable (α = .79) based on a widely adopted threshold of Cronbach’s alpha (Nunnally, 1967).

#### Person–Organization Fit

Person–organization fit was measured with a 3-item scale by Cable and DeRue (2002). The Cronbach’s alpha score for this scale (α = .75) indicated acceptable reliability.

Considering the theoretical overlap between organizational identification and fit, as well as their relatively high correlation (*r* = .58, *p* < .01), we calculated and compared the square root of average variance extracted (AVE) of both constructs with the value of the correlation between them to test for discriminant validity (Fornell & Larcker, 1981). We found that the square root value of the AVE of each construct, identification (√AVE = .69) and P-O fit (√AVE = .71), was higher than the correlation between them (.58). The latter indicates that the requirement of discriminant validity was met.

#### Job Dangerousness

Job dangerousness was assessed according to objective, dichotomous criteria given by the organization, based on functional area. Non-dangerous jobs were coded as 1 and dangerous ones as 2. Dangerous jobs (69 % of the sample) were defined as those requiring direct exposure to power lines and involving accidents resulting in severe injury (e.g., bursting a kidney or losing a hand), electrical shock or death, in areas like construction, technical maintenance and service of electrical equipment. Contrastingly, tasks that were not considered dangerous were performed in such departments of the company as logistics, administration, IT support, and finance.

#### Job Performance

Job performance was measured using a 3-item one-dimensional scale, first used in the Brazilian context by Lazzarini, Islam, and Mesquita (2011). Because this scale measured performance at the unit level in 3-year intervals, we adapted the items by changing the referent (Chan, 1998) and focusing on the last year’s performance to fit the current work context. The three items used are: *My individual performance over the last year was above average, My individual performance over the last year was excellent*, and *My group’s performance over the last year was excellent.* The measures of scale reliability were acceptable with Cronbach’s alpha score equal to .68 and composite reliability index exceeding the commonly adopted threshold of .7 (CR = .71) (Hair, Ringle, & Sarstedt, 2011). Additionally, we performed a confirmatory factor analysis of the construct using structural equation modeling (SEM) and confirmed that all the factor loadings were significant and above .50. Further, the results indicated that the one factor model fit the data well (RMSEA < 0.08, CFI > 0.95, CFI > 0.95, SRMR < 0.08). The latter in conjunction with the straightforward nature of the questions and their prior use in the Brazilian organizational context, provide sufficient evidence that the adaptation of the scale did not significantly change its psychometric properties.

#### Agreeableness and Neuroticism

Agreeableness and neuroticism were each measured using the 10-item scale by Goldberg et al. (2006). The Cronbach’s alpha for this study were .60 and .57 for neuroticism and agreeableness respectively, with composite reliability coefficients higher than .6 (CRagreeableness = .61 and CRneuroticism = .64). These values remain acceptable, even though the scale has demonstrated better properties in other studies. This might be due to the cultural context where our scale was administered, the relatively low educational level of the sample (36% of the participants attended only primary school and 51% had a high school diploma), and the specific context of the study; an electrical company in the interior of Brazil. Table 1, in the Results section, shows the means, standard deviations, and correlations between study variables.

### Data Analysis

To analyze our data, we used Process in SPSS (Hayes, 2013); a bootstrapping technique used for mediation and moderated mediation models. Following the recommendations from the most recent studies on mediation analysis (MacKinnon, 2008; Preacher & Hayes, 2004; Preacher, Rucker, & Hayes, 2007; Zhao, Lynch, & Chen, 2010), we tested Hypothesis 1 by first assessing the presence of an indirect effect of the independent variable (organizational identification) on the dependent variable (job performance) via the mediator (P-O fit), using a bootstrapping technique with 5,000 repetitions. We then evaluated if after controlling for the presence of the mediator, the direct effect of organizational identification on job performance was significant.

In contrast to the traditional, unidimensional approach to mediation by Barron and Kenny (1986), the method applied here does not exclude the existence of the mediating effect in the absence of a significant total effect of identification on performance. In fact, most recent studies argue that the condition necessary to clearly demonstrate and justify the presence of a mediating effect between two variables of interest, is to confirm the significance of an indirect effect between the two, regardless of the presence of “an effect to be mediated” (Hayes, 2009, 2013; Preacher et al., 2007; Zhao et al., 2010). This approach has been gaining increasing popularity in the organizational and psychology literature (e.g., Mostafa & Bottomley, 2018; Romani, Grappi, & Bagozzi, 2013; Santos, Zanette, Kwok, Heyman, & Lee, 2017) as it allows for more rigorous testing of mediating effects.

To test our 1st hypothesis, we used Process Model 4 designed to test simple mediation (Preacher et al., 2007). For Hypotheses 2, 3 and 4, we used Process Model 59 (Preacher et al., 2007) (also known as Total Effect Moderation Model, [Edwards & Lambert, 2007]) designed to test moderated mediation. Using Model 59 allowed us to test all the potential moderating routes of each moderator on the mediation established in Hypothesis 1, and thus confer conclusions on the nature of characteristics (personal vs. contextual) affecting identification and P-O fit work-related outcomes. Below we report the results of these analyses.

## Results

The results of Process Model 4 from the SPSS software that we used to test the 1^st^ mediating hypothesis, showed significant un-hypothesized effects of organizational identification on P-O fit (*b* = 0.45, *p* < .01) and of P-O fit on performance (*b* = 0.30, *p* < .01). Further, the results of the bootstrapping analysis confirmed the existence of a significant indirect mediating relationship between organizational identification and job performance through P-O fit (zero was not interceding in the value interval [0.0457, 0.2699]). The latter provides support for our 1st hypothesis. We also found a non-significant direct effect of organizational identification on job performance (*b* = -0.03, *p* = .69); a surprising result given the significant correlation in past studies (Riketta, 2005; van Knippenberg, 2000). According to the typology of mediation effects proposed by Zhao et al. (2010), we were able to find an *indirect-only mediation effect*, which occurs when the indirect path is significant while the direct path is not. The lack of direct effect in this study might be due to the particular nature of our sample which is recruited from a very different population to the white-collar one usually used in academic research. To test for possible reverse causality effects, we inverted the order of the mediation, testing the possible mediating effect of identification on the P-O fit—job performance relationship. The results were not significant (zero interceding in the value interval, [-0.1863, 0.1003]).

**Table 1:** Means, Standard Deviations, Correlations and Reliabilities

Variable	*M*	*SD*	1	2	3	4	5
1. Identification	5.13	1.45	(.79)				
2. P-O fit	5.65	1.13	.58**	(.75)			
3. Job performance	5.53	1.14	.12	.27**	(.67)		
4. Neuroticism	2.23	0.81	-.15	-.11	-.10	(.60)	
5. Agreeableness	5.89	0.76	.13	.21*	.22*	-.33**	(.57)
6. Dangerousness	1.70	0.46	-.29**	-.09	.08	.07	.06

*Note*. *N* = 109. Alpha coefficients in the diagonals. *SD* = standard deviation.

*p < .05. **p < .01.

To test our 2^nd^, 3^rd^ and 4^th^ hypotheses, we separately repeated the Process Model 59 to identify potential moderating effects of neuroticism, agreeableness and job dangerousness on the mediating effect reported above. A non-significant interaction was found between identification and agreeableness on P-O fit (*b* = -0.16, *p* = .07). Agreeableness was not found to moderate either the identification-performance or the P-O fit-performance relationships. Those results do not provide support for our 2nd hypothesis.

**Figure 1:**
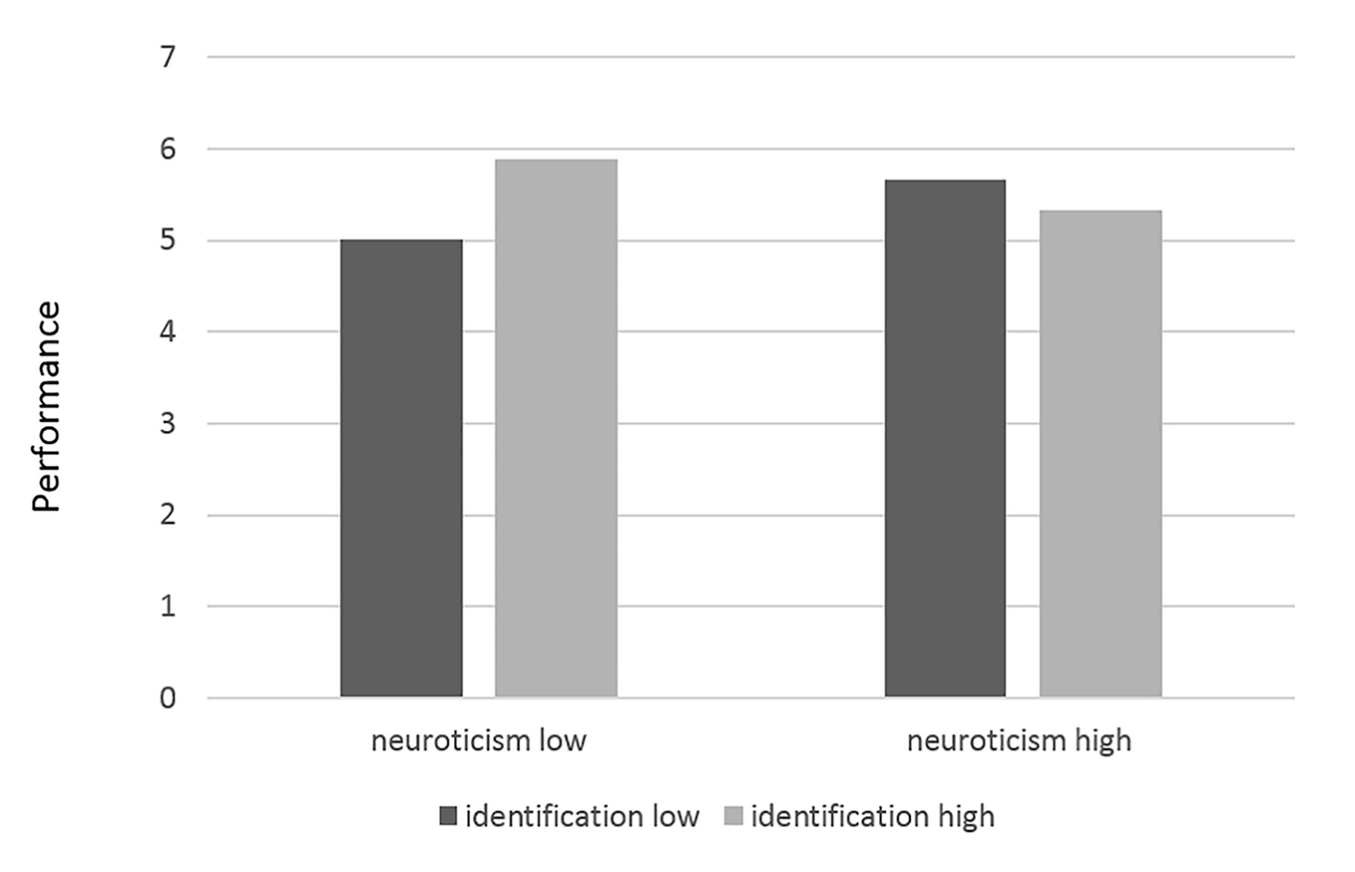
Interaction of identification and neuroticism on performance. *Note*. Black and grey depict low vs. high levels of identification respectively. Low vs. high levels of neuroticism are depicted in the x-axis.

Additionally, neuroticism significantly moderated the relationship between identification and job performance (*b* = -0.31, *p* = .03). However, neuroticism neither significantly interacted with identification to induce P-O fit nor with P-O fit to induce job performance. Pairwise comparisons revealed a positive significant main effect of identification on job performance for low (*b* = 0.33, *p* = .01), but not for highly (*b* = -0.13, *p* = .37) neurotic people. Those results provide support for our 3^rd^ hypothesis and are depicted in Figure 1.

**Figure 2:**
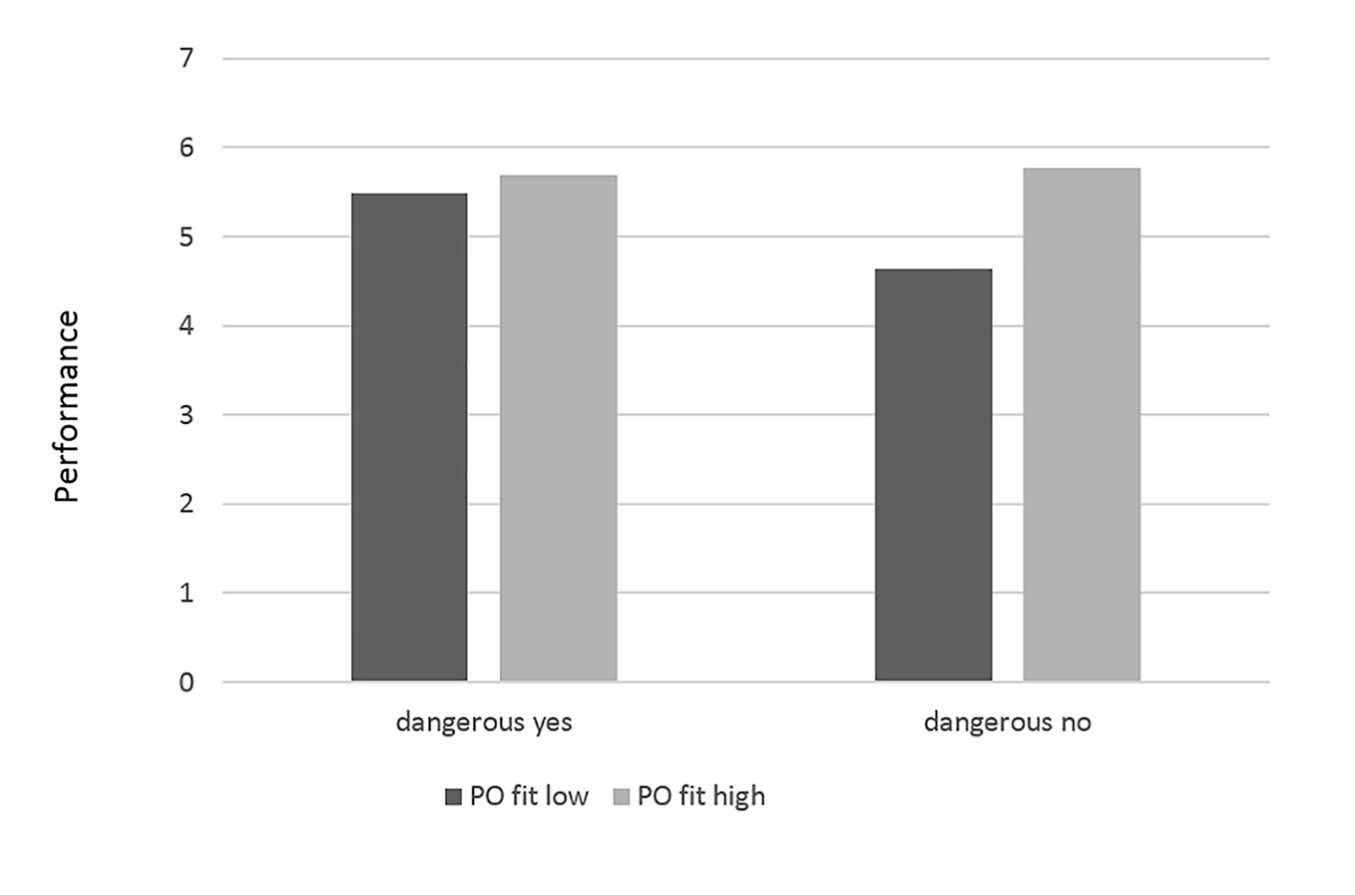
Interaction of dangerousness and P-O fit on job performance. *Note*. Black and grey depict low vs. high levels of P-O fit, respectively. Dangerous vs. non-dangerous environments are depicted in the x-axis.

**Figure 3:**
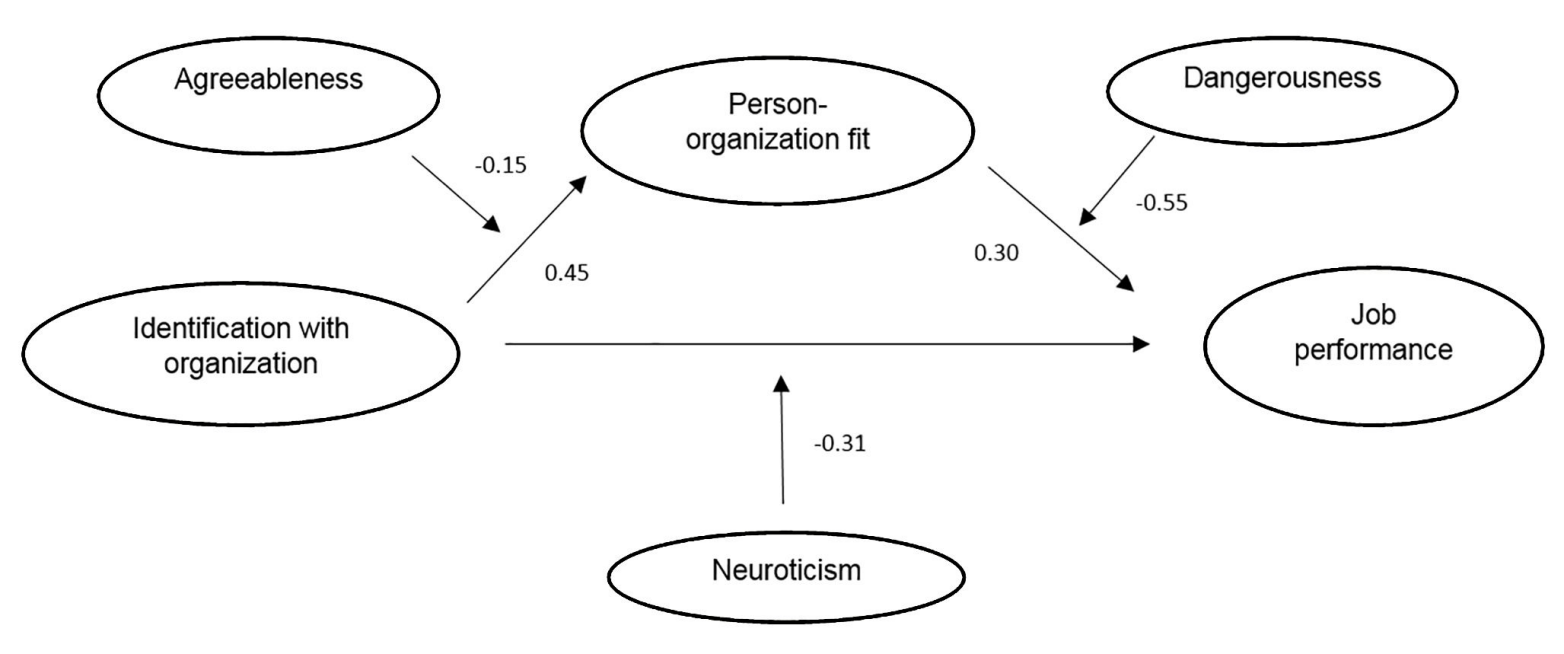
Reported effects of identification and P-O fit on performance.

Finally, dangerousness at work was only found to significantly moderate the relationship between P-O fit and job performance (*b* = -0.55, *p* = .04). Dangerousness was not found to guide the identification—performance relationship nor that between identification and P-O fit. Our results showed a positive main effect of P-O fit on job performance for non-dangerous jobs (*b* = 0.51, *p* < .01) but only a marginal effect for dangerous jobs (*b* = 0.21, *p* = .07). The latter results support our 4^th^ hypothesis and are depicted in Figure 2. The interaction results are shown in Table 2, while Figure 3 illustrates the results of our full model.

**Table 2:** Interaction Effects of Identification With Neuroticism, Agreeableness, and Dangerousness

Predictor Variable	Criterion Variables		
	Job Performance	Job Performance	Person-Organization Fit
Identification with organization	0.63		1.35
Neuroticism	0.73		
P-O fit		1.27*	
Dangerousness		1.88	
Agreeableness			1.05
Identification × Neuroticism	-0.31*		
Identification × Agreeableness			-0.16^†^
P-O fit × Dangerousness		-0.55*	

*Note.* The *b*s’ weights are reported after each step.

^†^*p* < .10. *p < .05.

## Discussion

Our results suggest that the relationship between organizational identification and job performance is enabled through P-O fit. Additional analyses showed that personality and contextual characteristics differently guide identification and P-O fit work-related outcomes. Personality appears more closely related to the former and context to the latter. Our results extend research treating personality and context as direct antecedents of identification and fit (Jansen & Kristof-Brown, 2006; Schneider, 2007). They also build on recent attempts to study the interactive effects of personality and states of perceived work alignment on work-related outcomes (Guan, Deng, Risavy, Bond, & Li, 2011; Resick et al., 2007). Similarly, our findings extend recent research citing positive reciprocal links between personal resources (stable individual differences) and work-related outcomes (Laguna, Razmus, & Zalinski, 2017).

Corroborating our intuition and previous research arguing on the self-based nature of identification (Postmes & Jetten, 2006) versus the context-laden nature of P-O fit (Edwards et al., 2006; Pratt, 1998), we provide evidence that links between identification and job performance are moderated by neuroticism, a trait reflecting different levels of emotional stability. Conversely, the P-O fit job performance relationship was only found to be guided by job dangerousness. Together those findings indicate that the underlying factors guiding the outcomes of P-O fit and identification on job performance differ in nature.

Our results also confirm previous findings citing indirect links between identification and job performance (Chen et al., 2013; van Knippenberg, 2000) and extend those. Particularly, van Knippenberg (2000) argued that identification only affects performance when high performance is viewed as a shared goal of the group and social identity in the group is strong. Similarly, recent research cites the role of work-group identification in enhancing the identification-performance relationship (Chen et al., 2013). Extending those findings, our study reveals an indirect relationship between identification and job performance, enabled through P-O fit. The latter suggests that fit perceptions might provide a conduit through which organizational identification leads to job performance. Proposing that organizational identification relates to work outcomes to the extent that it triggers more proximal relational states, namely P-O fit, we demonstrate how P-O fit can link cognition–organizational identification- with action–job performance-.

Further, by investigating the distinct underlying mechanisms shaping identification and P-O fit work outcomes, we show how the two may rely on distinct processes. We find that while personality, particularly neuroticism, does not directly lead to work outcomes (Barrick & Mount, 1991; Judge & Bono, 1999), it neutralizes the work-related outcomes of organizational identification. Namely, as expected, neuroticism appeared to negatively moderate the relationship between job identification and performance. As emotional instability, neuroticism may be undermining the positive emotional effects of identification that could affect job performance. However, agreeableness, curiously, did not appear to enhance the link between identification and P-O fit. One could argue that a ceiling effect might exist concerning the extent to which agreeableness can further enhance an already strong relationship between identification and P-O fit. Specifically, the effect of agreeableness may be stronger when the employees do not identify highly with their company such that their agreeable personality has a compensatory effect on the perceptions of fit between them and their organization. In fact, additional pairwise comparison analysis revealed a significant effect of agreeableness on P-O fit among low identifiers (*b* = 0.30, *p* < .05), yet weaker and insignificant for high identifiers (*b* = 0.90, *p* > .1). Future studies could explore this venue further to confirm and extend these results.

We also consider contextual characteristics, namely dangerousness, to further disentangle differential underlying processes guiding identification and P-O fit work outcomes. Our findings suggest that work dangerousness shapes the relation between subjective P-O fit and job performance, such that P-O fit work outcomes are weaker in dangerous job contexts. This finding extends past research arguing that dangerousness affects employee ability to complete job tasks and reduces performance at work (e.g., Pasupuleti et al., 2009; Price, 1997; Sousa-Poza & Sousa-Poza, 2000). We suggest that as a contextual factor, dangerousness primarily changes how people relate to their environment (P-O fit), rather than how they define themselves as members of their group (organizational identification).

Taken together, our model indicates that work-related outcomes of organizational identification and P-O fit are driven by distinct factors. Particularly, we propose that one’s feelings of belongingness and oneness with the working context are personality and not context-driven, while work effects of subjectively perceived organizational congruence are context and not personality-driven. Our results are in line with previous literature discussing identification as a mainly self-focused process (Postmes & Jetten, 2006) and subjective P-O fit as a largely contextual one (Edwards, 2008; Pratt, 1998).

Considered globally, our findings extend research studying direct identification and P-O fit job outcomes (Jansen & Kristof-Brown, 2006; Schneider, 2007) by demonstrating how individual and contextual characteristics can differently drive their respective work-related processes in an underlying manner. Similarly, our findings complete and extend research citing linkages between behavioral characteristics and states of perceived employee alignment in predicting job-related outcomes like job performance (Nicol et al., 2011; Schneider, 2007; Vignoles et al., 2006). Further, our results contribute to debates on the role of understudied personality traits on organizational identification’s job-related outcomes (e.g., Barrick & Mount, 1991; Hogan & Holland, 2003; Mount et al., 1998). More broadly, we build on literature citing mutual links between personal resources (such as self-efficacy) and work-related outcomes, such as job engagement (Laguna et al., 2017). Last but not least, responding to recent calls for research on the individual and contextual factors driving the effects of organizational alignment states (Kristof-Brown, 2005), we contribute to existing discussions around the differential mechanisms guiding organizational identification and P-O fit (Edwards, 2008; Pratt, 1998). In doing so, we enhance clarity on the conceptual differences between the two constructs.

### Limitations and Future Research

Some limitations to our study remain. First, measuring job performance and personality characteristics with a self-report measure, increases the risk of common method bias and should be interpreted with caution (Podsakoff, MacKenzie, Lee, & Podsakoff, 2003). However, since our subjects’ job categories were incommensurable, objective criteria of performance did not exist across categories, making subjective criteria more comparable. Precedent for this usage is drawn from past literature (Cook, Young, Taylor, & Bedford, 2000; Dabke, 2014). Further, interaction effects are less likely to be subjected to common method effects (Podsakoff et al., 2003) and the main effects of both variables have been established in past literature (Ashforth et al., 2008; Edwards, 2008; Jansen & Kristof-Brown, 2005). Lastly, additional robustness comes from the fact that dangerousness was an objectively measured job characteristic, while the personality instrument used has been widely validated in previous research (Hunter et al., 2013). Additionally, P-O fit was measured with a 3-item scale potentially disregarding the impact of specific factors shaping employees’ perceptions of subjective fit at work. Building on our findings, future research could use longer measures of P-O fit to account for more elements shaping such perceptions of work-place alignment.

Further, the lack of a longitudinal study design limits our ability to draw conclusions about causality. Future research could extend our findings. Finally, a relatively small sample size may limit our results’ generalizability. Given that the survey was distributed by the HR of the company, its extent was limited, and we could not engage in snowball sampling or global surveying at the company. Future research could use bigger samples to replicate those results.

In addition, although we have performed additional analyses testing the psychometrical properties of the job performance scale by computing its reliability and performing confirmatory factor analysis, additional tests of construct validity can be performed. For instance, future studies can probe into the criterion validity of the scale and test its ability to predict some well-established job performance outcomes, such as pay-related performance measures or career satisfaction (Welbourne, Johnson, & Erez, 1998).

Given the extent of scholarly discussion around identification and P-O fit, the integration of these constructs within a framework simultaneously considering both personal and situational moderating variables, seems crucial to the literature. Proposing that organizational identification and P-O fit may be operating on different parts of the motivational process (personal engagement vs. situational alignment), we contribute to the debate on the theoretical overlap between these two constructs and delineate conceptual differences between the two. Future research could build on our findings and look for other self and context-related constructs differently affecting organizational identification and P-O fit.

Further, given our current focus on psychological, cognitive and behavioral factors, future research could investigate the role of emotions in guiding the dynamics of our proposed model. For instance, it would not be unexpected to see a neutralizing effect of positive emotions on the way neuroticism drives organizational identification work outcomes. Finally, yet importantly, one could test whether objective situational characteristics (i.e., dangerousness) and P-O fit can jointly predict emotional work-related responses, such as trust or anxiety.

#### Managerial Implications

In terms of managerial implications, untangling the effects of identification and P-O fit can be useful in informing organizational policies around worker motivation. Specifically, to the extent that companies encourage employees to “wear the hat” of the enterprise, identifying with its culture or brand, it is important to note that such categorization mechanisms may rely on more proximal forms of alignment to be effective, and that such effects are likely to vary by personality and job type. In the absence of fit perceptions, identification alone may be insufficient to provide desired outcomes.

Similarly, our study suggests that while managers who supervise teams working in safe environments should enhance the employees’ subjective fit with the organization in order to increase their performance, such efforts are not likely to be effective in dangerous contexts. In other words, initiatives enhancing alignment of personal and team goals (Downes, Kristof-Brown, Judge, & Darnold, 2017) are more likely to promote P-O fit and job performance in administrative departments rather than in areas where the workers are exposed to risks of injury or death. Therefore, knowing that dangerousness prevents employees from performing better, even under conditions of high P-O fit, managers should minimize employee exposure to dangerous conditions or try to counterbalance them with the provision of health protection and safety measures (e.g., generous health insurance packages, etc.).

In addition, the positive indirect relationship of organizational identification on job performance through P-O fit has implications for managers of employees, who are hired despite potential incompatibility between their and the company’s values. Our findings show that such employees are able to improve their alignment with the company, and consequently perform better, once they start to identify with the company. This can be achieved through team building activities, positive communication efforts and favorable climate within the team (Albert et al., 2000; Smidts, Pruyn, & Van Riel, 2001).

Also, having shown that neuroticism underlies identification’s effects on job performance, managers in charge of recruitment should consider candidates’ personality profiles. In particular, they should be aware that highly neurotic individuals tend to underperform even under conditions of high identification. Thus, they may either give precedence to the emotionally stable candidates or ensure the provision of psychological support to particularly neurotic individuals.

### Conclusion

The current paper studies the different underlying processes guiding work-related outcomes of organizational identification and P-O fit. Our model proposes that neuroticism underlies job outcomes of organizational identification, whereas dangerousness at work shapes the relationship between perceptions of P-O fit and performance. Our results support theory citing identification as a self-oriented process, whose work-related effects are largely shaped by personality characteristics. On the other hand, we find that as a mainly context-dependent variable, P-O fit work outcomes are contingent on contextual characteristics. If considered by managers, our results can help to enhance employee and work environment management and allow employees to achieve the desired performance levels across various working conditions.
